# Evidence for conserved post-transcriptional roles of unitary pseudogenes and for frequent bifunctionality of mRNAs

**DOI:** 10.1186/gb-2012-13-11-r102

**Published:** 2012-11-15

**Authors:** Ana C Marques, Jennifer Tan, Sheena Lee, Lesheng Kong, Andreas Heger, Chris P Ponting

**Affiliations:** 1 MRC Functional Genomics Unit, University of Oxford, Department of Physiology, Anatomy and Genetics, South Parks Road, Oxford, OX1 3PT, UK; 2 University of Oxford, Department of Physiology, Anatomy and Genetics, South Parks Road, Oxford, OX1 3PT, UK

## Abstract

**Background:**

Recent reports have highlighted instances of mRNAs that, in addition to coding for protein, regulate the abundance of related transcripts by altering microRNA availability. These two mRNA roles - one mediated by RNA and the other by protein - are inter-dependent and hence cannot easily be separated. Whether the RNA-mediated role of transcripts is important, *per se*, or whether it is a relatively innocuous consequence of competition by different transcripts for microRNA binding remains unknown.

**Results:**

Here we took advantage of 48 loci that encoded proteins in the earliest eutherian ancestor, but whose protein-coding capability has since been lost specifically during rodent evolution. Sixty-five percent of such loci, which we term 'unitary pseudogenes', have retained their expression in mouse and their transcripts exhibit conserved tissue expression profiles. The maintenance of these unitary pseudogenes' spatial expression profiles is associated with conservation of their microRNA response elements and these appear to preserve the post-transcriptional roles of their protein-coding ancestor. We used mouse *Pbcas4*, an exemplar of these transcribed unitary pseudogenes, to experimentally test our genome-wide predictions. We demonstrate that the role of *Pbcas4 *as a competitive endogenous RNA has been conserved and has outlived its ancestral gene's loss of protein-coding potential.

**Conclusions:**

These results show that post-transcriptional regulation by bifunctional mRNAs can persist over long evolutionary time periods even after their protein coding ability has been lost.

## Background

Transcript levels can be regulated in a spatiotemporal manner both transcriptionally and post-transcriptionally. Recently, a new layer of post-transcriptional expression regulation was revealed that involves competition among transcripts for binding to specific microRNAs (miRNAs; 22 to 25 nucleotide noncoding RNAs) [1-3]. Negative regulation of mRNA levels by miRNAs appears to be widespread among eukaryotes and involves the recognition and binding of mature miRNAs to miRNA response elements (MREs) that are often located in the 3' untranslated regions of target mRNAs [4-6]. miRNAs are largely preserved in animal evolution [7] and mutations in either MREs or miRNAs have been associated with gene expression changes leading to phenotypic differences (for example, [8,9]; reviewed in [4,10,11]). While these observations imply that miRNAs have considerable functional importance, their experimental deletion rarely results in overt phenotypes and the effects on gene expression of altered miRNA levels are often only modest [12]. A miRNA can regulate large numbers of transcripts [13,14], and target recognition is thought to result in decreased miRNA levels [4]. Consequently, transcripts can indirectly alter the abundance of other transcripts if they share MREs; transcripts that engage in such post-transcriptional crosstalk have been termed 'competitive endogenous RNAs' (ceRNAs) [15].

Several protein-coding transcripts have been shown to act as ceRNAs [1,16]. The protein-coding and miRNA-mediated roles of mRNAs are not independent: targeting of miRNAs to a transcript's MREs can result in decrease levels of its encoded protein and mRNA abundance will regulate, through competition for miRNAs, the levels of other transcripts [1,16,17]. It is this coupling between RNA- and protein-dependent functions of a transcript that renders the biological importance and implications of ceRNAs so difficult to determine. As a result, it has remained unclear whether a transcript's MREs might be sufficiently important for its miRNA decoy function to act autonomously of its protein-coding capability - for example, by conferring robustness to transcriptional networks or by buffering genetic noise [12]. Noncoding transcripts have also been shown to function as competitive endogenous RNAs [2,18]. Whether these noncoding ceRNAs have other functions - for example, with additional transcriptional or chromatin regulation roles [19] - remains to be established.

To date, studies have only focused on the post-transcriptional roles of transcribed pseudogenes that share MREs with their duplicated homologous transcripts (reviewed in [20,21]). *PTENP1*, for example, is a processed (that is, retroduplicated) pseudogene that acts as a ceRNA by modulating the expression level of its parental gene *PTEN*, a known tumor suppressor, with which it shares several predicted MREs [11]. Complete or partial deletion of *PTENP1 *occurs in several human tumors and is associated with decreased expression of *PTEN*, which in turn is expected to result in cell proliferation [1,22]. *PTENP1*, and other transcribed pseudogenized gene duplicates [11], provide important insights into the origin of new competitive endogenous RNAs by gene duplication that establish indirect transcript-transcript interactions between homologous, including parent-retroduplicated, gene pairs. Nevertheless, because most ceRNA networks will involve crosstalk between multiple, often non-homologous, transcripts [3,16,20], we have as yet little information on the relative importance of miRNA-mediated roles of mRNAs - for example, whether these are subordinate to their presumed primary, often protein-coding, functions. Only by addressing this question will we be able to understand fully the contribution of such post-transcriptional regulatory mechanisms to animal transcriptional regulation.

To separate the RNA- from the protein-mediated actions of mRNAs, we identified genes that each lost their role as a protein-coding message in the rodent lineage (including mice and rats), and compared them to their human orthologs that each retained protein-coding capability; these are termed unitary pseudogenes. In contrast to the high number of duplicated and retroduplicated pseudogenes (of which *PTENP1 *is an example) in mammalian genomes [23,24], unitary pseudogenes are rare. Unitary pseudogenes derive from the lineage-specific acquisition of disrupting mutations in the coding sequences of genes [25-28]. Some pseudogenes have been observed to be transcribed and can function as RNAs, and thus might be considered to be *bona fide *genes [29].

**Figure 1:**
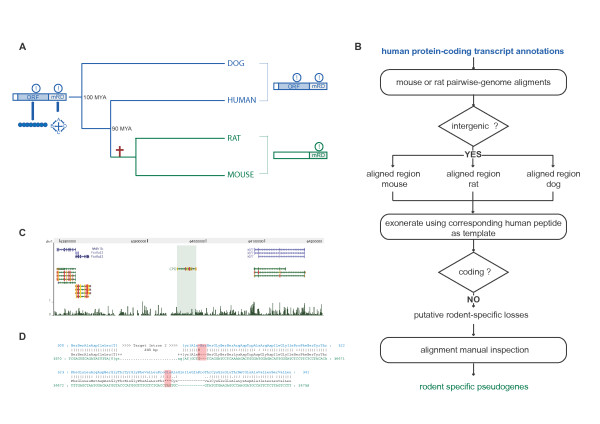
**Identification of rodent-specific unitary pseudogenes**. **(a) **Evolution of rodent-specific pseudogenized bifunctional transcripts. An ancestral transcript encoding a functional (shown by the exclamation mark) protein (vertical lines indicate an intact open reading frame (ORF)) and with a role as a miRNA decoy (mRD) has accumulated one or more mutations disabling its ORF (red cross) on the lineage leading to rodents. As a result the mouse or rat unitary pseudogene (green) has lost its coding potential while retaining its function as a competitive endogenous RNA while both functions are conserved in the protein-coding loci (blue) in human or dog. MYA, million years ago. **(b) **Flowchart for identifying rodent-specific losses of human protein-coding genes. **(c) **Genome browser view of the transmap annotation (green block) for the mouse unitary pseudogene of human *carboxypeptidase O *(*CPO*). This unitary pseudogene is located in an intergenic region of the mouse genome (chr1:63,950,645-63,982,545, mm9). Protein-coding genes downstream (*Fastkd2*) and upstream (*Klf7*) of this sequence are conserved between mouse and human as illustrated by the overlap between transmap and the UCSC protein coding gene annotations (in blue). The mammalian conservation track on the bottom (UCSC genome browser) shows the degree of placental mammal base pair conservation (20 species). **(d) **Portion of pairwise alignment between the human CPO peptide (blue) and the syntenic sequence in mouse (green). Highlighted in red are a frame shifting deletion at the start of exon 3 and the resulting downstream premature stop codon.

Investigations of transcribed unitary pseudogenes allow us to dissect their miRNA-mediated roles away from their ancestral protein-coding functions (Figure 1a). In-so-doing, we are able to consider whether these noncoding roles have been conserved between humans and rodents since they last shared a common protein-coding ancestor approximately 90 million years ago. Conservation of ancestral post-transcriptional miRNA decoy functions would imply that the miRNA-mediated interactions between transcripts are biologically relevant, linking, for example, functionally related genes [30] or serving as a post-transcriptional buffer of gene expression.

## Results and discussion

### A stringent catalogue of rodent-specific unitary pseudogenes

We started by identifying 758 human protein coding gene transcripts whose alignments within the conserved syntenic region [28] of the mouse genome had no overlap (by 1 base pair or more) with mouse protein-coding gene annotations (ENSEMBL build 67). We performed exhaustive 6-frame translated pairwise [31] alignments between the 758 human polypeptide templates and their regions of conserved synteny (extended by 5 kb upstream and downstream) in the mouse and rat genomes and that of dog (used as an out-group) to identify mammalian protein-coding genes that were lost specifically in mouse and rat (that is, rodent-specific unitary pseudogenes). In the three species, we classified the sequence corresponding to the best alignment either as a unitary pseudogene or else as a conserved gene [32] (Figure 1b). Using this approach we predicted 48 human protein-coding genes (Additional file 1) that have conserved their protein-coding potential in dog and that are unitary pseudogenes in mouse and rat. Predictions were visually inspected to ensure that: 1) frame-shifting indels or premature stop codon mutations were specific to both rodents; and 2) chromosomal gene order for genes immediately upstream and downstream of the lineage-specific unitary pseudogene was conserved in all four species [33,34].

The 48 rodent-specific unitary pseudogenes are comparable in number to primate unitary pseudogenes [25,28], suggesting that proteins are lost at similar rates in the rodent and primate lineages. The human carboxypeptidase O (*CPO*) gene is one example whose mouse or rat ortholog is a unitary pseudogene (Figure 1c, d). It maps to an unannotated region of the mouse genome (Figure 1c) whose pairwise human-mouse alignment reveals two disabling mutations (Figure 1d; Additional file 2) that are predicted to result in a truncated open reading frame in the rodent *CPO *orthologous sequence.

### Transcribed mouse unitary pseudogenes do not encode conserved peptides

We used publicly available RNAseq data across six adult mouse tissues to identify rodent-specific unitary pseudogenes that are transcribed in these mouse tissues [35]. We were able to assemble transcripts, or fragments thereof, using Cufflinks [36] (Materials and methods; Additional file 3) for 17 (35%) of these rodent-specific unitary pseudogenes. We then validated the expression of 16 of these 17 unitary pseudogenes using a second RNAseq dataset that includes data from 19 mouse tissues/cells and that has a higher sequencing depth [37]. The failure to validate expression of the seventeenth unitary pseudogene (*LCNL1*) may reflect this transcript's relatively low expression level. This unitary pseudogene has a H3K4me3 mark [37], an indicator of transcription initiation, in the cerebellum, one of the three tissues in which this transcript was initially identified.

Interestingly, the second RNA-seq dataset supported the expression of a further 15 unitary pseudogenes (Additional file 4), suggesting that at least 65% of the rodent-specific unitary pseudogenes are expressed.

Concentrating on the 17 unitary pseudogenes with expression evidence from both datasets [35,37] (Additional file 3), we next considered whether their *de novo *assembled transcripts (Additional file 3) encode proteins. The median score associated with codon substitution frequencies between mouse and rat [38] for unitary pseudogene transcripts was found to be -16.1, which is smaller than zero, as expected for noncoding regions [38], and substantially smaller than the corresponding score (25.4) for 1,000 randomly selected protein-coding gene transcript fragments matched in size to the unitary pseudogene transcripts (*P *< 10^-16^, two-tailed Mann-Whitney test; Additional file 5). The nucleotide substitution pattern between mouse and rat indicates that these transcribed unitary pseudogenes are unlikely to encode a conserved protein. We next estimated the coding potential of mouse unitary pseudogene transcripts using CPC [39], which considers the length of putative open reading frames and their homology to known mammalian proteins. Only 3 out of the 97 transcripts longer than 200 nucleotides were annotated as coding. As expected, the putative open reading frames in these transcripts are homologous but incomplete due to the accumulation of deleterious mutations to the protein-coding ortholog in humans. Furthermore, the fraction of unitary pseudogene transcripts annotated as coding is over 20 times smaller than found for 1,000 randomly selected protein-coding transcript fragments with matching size, a highly significant difference (656/1,000, *P *< 10^-4^, two-tailed Fisher's exact test). These findings indicate the 17 transcribed unitary pseudogenes are unlikely to have retained their protein-coding capacity.

### miRNA decoy functions are preserved after loss of protein-coding potential

We next compared the expression level (FPKM values; total number of fragments per kilobase of sequence per million reads mapped) and tissue specificity (maxT*_S_*) between unitary transcribed pseudogenes and protein-coding genes (Additional file 6; Materials and methods) across six adult mouse tissues [35]. The median expression of transcribed unitary transcripts (median FPKM = 0.56; Additional files 6 and 7) is significantly lower than that of protein-coding genes (median FPKM = 2.91, *P *< 10^-4^, two-tailed Mann-Whitney test; Additional files 6 and 7). In contrast, mouse unitary pseudogenes are as ubiquitously expressed in adult mice (Materials and methods) as protein-coding genes (median maxT*_S _*= 0.26 and 0.28, respectively; *P *= 0.42, two-tailed Mann-Whitney test; Additional files 6 and 7).

If a transcribed mouse unitary pseudogene has maintained the decoy roles of its protein-coding ancestral transcript (Figure 1a), one expects that its tissue expression profile would be conserved and thus shared with its protein-coding human ortholog. Indeed, we found (see Materials and methods) that these 17 mouse unitary pseudogene-human gene ortholog pairs are more highly correlated (median Pearson correlation coefficient = 0.20) than randomly sampled pairs of protein-coding genes (median Pearson correlation coefficient = 0; Additional file 8) in their expression profiles. This implies that the relative expression levels of these unitary pseudogene transcripts, across the adult tissues we tested, were preserved, at least in part, after loss of protein coding capability.

**Figure 2:**
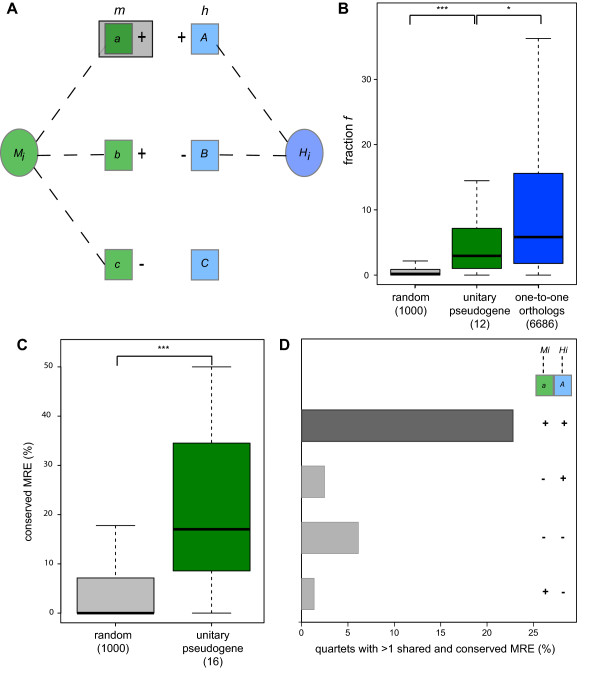
**Conserved genetic interactions of transcribed unitary pseudogenes**. **(a) **We estimated the correlation in expression between orthologous mouse (green) or human (blue) loci (*M_i _*and *H_i_*, respectively, ovals) and other protein-coding genes (rectangles) annotated in mouse or human genomes. We retained only genes exhibiting significantly (empirical *P*-value < 0.05) positive (+) and negative (-) correlation with *M_i _*or *H_i _*tissue expression (dashed line). We identified all mouse genes (for example, 'a' boxed) whose expression profiles are significantly correlated with that of *M_i_*, and whose human one-to-one ortholog (for example, 'A') is also significantly correlated in the same direction with *H_i_*. **(b) ***f *is the fraction of mouse genes, *m*, that are significantly correlated in expression with a unitary pseudogene, *M_i_*, whose human orthologs are also significantly correlated in expression (and in the same direction, either positively or negatively) with *H_i_*, the human ortholog of *M_i_*. *f *for human protein-coding gene and mouse unitary pseudogene pairs (green) is significantly higher (***P < 0.001) than for random non-orthologous mouse and human gene pairs (grey) and significantly lower (*P < 0.05) for mouse and human one-to-one orthologs. No constitutively expressed exons (required to measure gene expression) were identified for *EXD3, THAP9, RNF175, DBF4B *and *ZBED*. Hence these genes were not considered in this analysis. **(c) **Unitary pseudogenes (green) share significantly (***P < 0.001) more MREs with their human protein-coding ortholog 3' UTR than random pairs of mouse and human protein-coding genes (grey). MRE predictions were not available for *ZBED5 *and this locus was not considered in this analysis. **(d) **Twenty-two percent of conserved significantly positively (++) correlated quartets (*Mi*-*A*-*a*-*Hi*, dark grey) share at least one MRE for the same miRNA family across the four loci. This is a significantly higher fraction than found for quartets that are significantly negatively correlated (--) or significantly correlated (+- and -+) in different directions (light grey).

We then looked for the conservation of post-transcriptional regulatory networks involving the mouse unitary pseudogene or its human protein-coding gene ortholog. To do this, we asked whether a gene pair whose tissue expression values are significantly positively correlated in mouse are also significantly positively correlated in their expression in human. For a mouse gene, *M_i_*, we identified a set of mouse genes, *m*, whose expression is significantly correlated (empirical *P*-value < 0.05) with *M_i_*. Similarly, for the human one-to-one orthologous gene, *H_i_*, of mouse *M_i_*, we identified the set of human genes, *h*, whose expression is significantly correlated with *H_i_*'s expression values. Finally, we calculated the fraction, *f_i_*, of all mouse genes in set *m *that have human one-to-one orthologs in set *h *with positively correlated expression levels (Figure 2a). When *M_i _*and *H_i _*are an orthologous pair of protein-coding genes, the median fraction *f *of *h *with *m *is 5.5% (Figure 2a). When *M_i _*is a mouse transcribed unitary pseudogene and *H_i _*is its orthologous protein-coding gene, the median fraction *f *is 1.0% (*P *< 0.05, two-tailed Mann-Whitney test; Figure 2b). When mouse *M_i _*and human *H_i _*genes are randomly paired, the median fraction *f *is significantly smaller (median = 0, *P *< 4 × 10^-8^, two-tailed Mann-Whitney test; Figure 2b). This analysis provides evidence, albeit at lower levels than for mouse-human protein-coding pairs, for the conservation of expression for orthologous mouse unitary pseudogene-human gene pairs. The conservation of their expression patterns is likely due to the preservation of their ancestral regulatory elements. We hypothesized that this conservation reflects, at least in part, the preservation of post-transcriptional networks involving these rodent-specific unitary pseudogene transcripts after the loss of their ancestral protein-coding capability (Figure 1a).

If two transcripts regulate each other's expression post-transcriptionally by competing for miRNA binding, we would expect their expression to be positively, rather than negatively, correlated [33]. We identified 19,703 mouse unitary pseudogene-mouse gene pairs whose expression profiles are positively correlated. Of these pairs, 1,340 (6.8%) have human orthologs whose expression profiles are also positively correlated (hereafter, termed conserved positively correlated quartets). In contrast, of the 13,579 negatively correlated pairs, a significantly lower proportion (607/13,579, 4.4%, two-tailed chi-square test, *P *< 10^-4^) have human orthologs whose expression profiles are also negatively correlated. This higher level of preserved positive correlation is consistent with these transcripts forming part of conserved ceRNA networks.

We next investigated whether this conservation of post-transcriptional networks is mediated by the preservation of orthologous miRNAs and their cognate MREs in mouse and human orthologous 3' untranslated regions (3' UTRs). We found that almost a fifth (17%) of MREs predicted in mouse unitary pseudogene transcripts are also identified in the 3' UTR of their human protein-coding orthologs. This is significantly higher than expected based on shared MREs between random pairs of mouse and human protein-coding non-orthologous 3' UTRs (0, *P *< 10^-4^, two-tailed Mann-Whitney test; Figure 2c). Next we identified MREs predicted in mouse unitary pseudogenes (*M_i_*) that were shared with protein-coding genes with which they had correlated expression. We considered 1,340 quartets of mouse and human loci that contain one of the mouse unitary pseudogenes (*M_i_*) and its human protein-coding ortholog (*H_i_*), and a pair of mouse and human orthologous genes that are each positively correlated in expression profile with *M_i _*or *H_i _*(Figure 2a and see above). For 22% of these conserved positively correlated quartets at least one MRE predicted in *M_i_*, the mouse unitary pseudogene, was also predicted in the 3' UTRs of each of the three other genes in the quartet. This is a significantly higher fraction (1.3 to 6.1%, *P *< 10^-3^, two-tailed Fisher's exact test; Figure 2d) than found for gene quartets associated either with significant negative expression correlations or with significant correlations that are in opposing directions in mouse and human (Figure 2d).

Taken together, these results suggest transcribed rodent-specific unitary pseudogenes frequently conserve their protein-coding ancestor's post-transcriptional roles and networks, and act as competitive endogenous RNAs.

### *BCAS4 *pseudogene, *Pbcas4*, is a conserved competitive endogenous RNA

**Figure 3:**
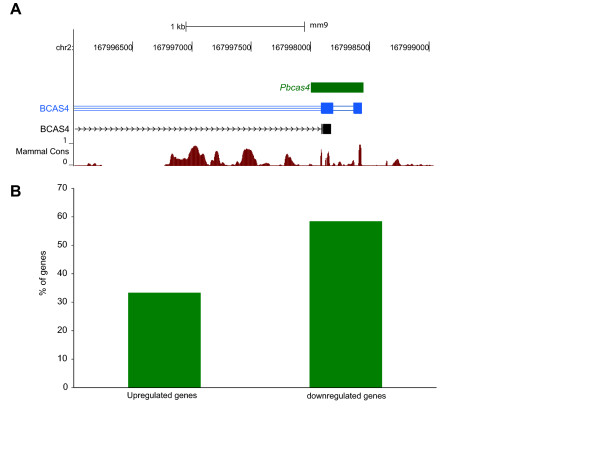
**The conserved function of *Pbcas4 *as a competitive endogenous RNA**. **(a) **Genome browser view of the transmap annotation (blue track) for the *human breast carcinoma amplified sequence 4 *(*BCAS4*) unitary pseudogene in mouse, *pbcas4*. The tBLASTn alignment available from UCSC between the BCAS4 peptide and the mouse genome is in grey. The full length transcript in N2A (green) is transcribed from chr2:167,998,005-167,998,447 (mm9) and aligns to the 3' UTR region of the human *BCAS4 *gene The mammalian conservation track on the bottom (UCSC genome browser) shows the degree of placental mammal base pair conservation (20 species). **(b) **Knock-down of *Pbcas4 *expression leads to down-regulation of protein-coding genes that have conserved human and mouse MREs for miR-185/882 and miR-665 families. The percentage of genes that have a conserved (mouse and human) MRE for either miR-185/882 or miR-665 (Y-axis) is 33% and 58% for genes up- and down-regulated, respectively, upon *Pbcas4 *knockdown.

Our computational findings predict that mouse transcribed unitary pseudogenes are ceRNAs and that this post-transcriptional regulator function is ancestral and shared with their orthologous human protein-coding genes. To investigate this prediction, we chose *Pbcas4*, one of the 17 mouse unitary pseudogenes, for further study on the basis of its ubiquitous and relatively high expression in mouse adult tissues (Additional file 7). Mouse *Pbcas4 *is the transcribed unitary pseudogene of human *BCAS4 *(Figure 3a), which has protein-coding orthologs conserved from diptera to early branching vertebrates. The full-length transcript of mouse *Pbcas4*, as determined using rapid amplification of cDNA ends (RACE) in neuroblastoma cells (N2A), corresponds only to the human *BCAS4 *3' UTR sequence (Figure 3a).

To investigate the transcriptome-wide effect of reduced abundance of *Pbcas4 *transcripts in N2A cells, we designed and cloned short hairpin sequences (shRNAs; Additional file 9) specific to target *Pbcas4 *and used microarray technology to estimate transcript expression change. Decreased amounts of *Pbcas4 *led to the differential expression of 165 genes (Materials and methods), of which a significant majority (96) were down-regulated (*P *< 0.05, binomial test). If *Pbcas4 *has a conserved function as a competitive endogenous RNA, human orthologs of these 96 down-regulated protein-coding genes would be expected to exhibit positively correlated gene expression with *BCAS4*, the human protein-coding ortholog. Indeed, this was found to be the case: we identified 41 such genes, whereas 28 genes would be expected simply by chance (46% increase; *P *< 10^-4^, binomial test; Materials and methods).

Of the 12 MREs predicted in the full-length *Pbcas4 *transcript in N2A cells, 2 (miR-185/882 and miR-665) are also predicted to bind the human orthologous *BCAS4 *3' UTR. Mouse genes containing predicted MREs for either miR-185/882 or miR-665 that are predicted also in their human ortholog are nearly twice as likely (1.7-fold increase, *P *< 0.02, Fisher's exact test; Figure 3b) to be among the genes down-regulated upon *Pbcas4 *knockdown than among those that are up-regulated. This finding is consistent with *Pbcas4 *sharing a miRNA decoy function with its human protein-coding ortholog.

**Figure 4:**
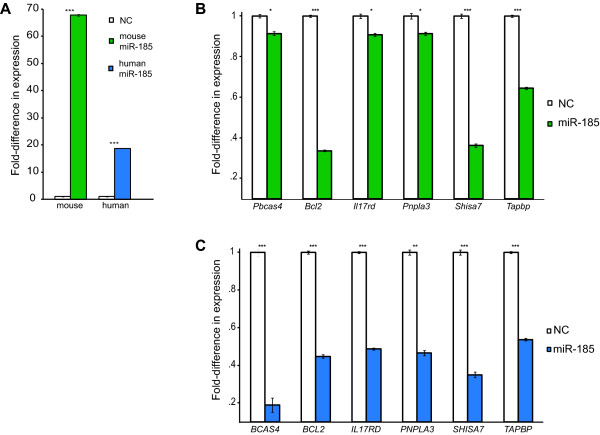
**miR-185 mediates conserved crosstalk between *Pbcas4 *and *BCAS4 *and protein-coding gene ceRNAs in mouse and human**. **(a-c) **An increased concentration of miR-185 in mouse and human neuroblastoma cells (N2A and SH-SY5Y cells, respectively) (a) leads to significant down-regulation of *Pbcas4, BCAS4 *and five mouse (b) and human (c) orthologous pairs that were predicted to compete for binding of miR-185. Asterisks indicate significance the level of the comparison (*t*-test) between the expression of target transcripts after transfection of negative control (set to 1) and miR-185 mimic (**P < 0.01; ***P < 0.001, NC; *P *> 0.01).

To test this hypothesis, we selected five protein-coding genes (*Bcl2, Ill7rd, Pnpla3, Shisa7 *and *Tapbp*; Additional file 11) whose expression was significantly down-regulated by reduced expression of *Pbcas4 *and whose mouse and human protein-coding gene orthologous pairs are both predicted to have miR-185/882 and miR-665 MREs in their 3' UTRs. We tested by quantitative RT-PCR for the expression levels of *Pbcas4 *and the 5 protein-coding genes, 24 hours after transfection of mouse neuroblastoma cells with mimics of miR-185. miR-882 is not expected to be expressed in N2A cells [40]. We chose not to test miR-665 since, unlike miR-185, its mature sequence differs, by a single nucleotide, in human and mouse. Consistent with the expression of *Pbcas4 *and the 5 protein-coding gene candidates being post-transcriptionally regulated by miR-185, a 68-fold increase in this miRNA resulted in significantly reduced abundance of mouse *Pbcas4 *and each of the 5 predicted protein-coding transcript targets (*P *< 10^-4^, ANOVA, mean fold-change in expression 0.68; minimum fold-change in expression 0.34; Figure 4a, b).

To test whether post-transcriptional regulation by this miRNA is conserved in humans, we similarly transfected the miR-185 miRNA mimic in human neuroblastoma cells (SH-SY5Y). It was striking that transcript abundance of *BCAS4 *and of each of the five human genes (*BCL2, ILL7RD, PNPLA3, SHISA7 *and *TAPBP*) was also significantly reduced upon a 19-fold increase in miR-185 level (*P *< 10^-4^, ANOVA, mean fold-change in expression 0.41; minimum fold-change in expression 0.19; Figure 4a, c). These results again indicate that mouse *Pbcas4 *has retained the post-transcriptional role of its protein-coding gene ancestor.

## Conclusions

Competition for miRNA-binding between transcripts with shared MREs has recently been demonstrated in animals and plants. miRNA-mediated crosstalk with many non-homologous mRNAs as participants is likely to be complex and to contribute substantially to the regulation of a transcript's cellular concentration [3,15]. However, it has remained unclear whether a transcript's role as a miRNA 'decoy' is crucial for either its molecular or organismal function, or whether the biological importance of the decoy role is marginal, owing to the promiscuity of miRNA-binding. To address this issue, we considered 48 rodent-specific unitary pseudogenes that have lost their protein-coding capability during rodent evolution. Consistent with previous reports, a substantial fraction of unitary pseudogenes are expressed despite apparently lacking an open reading frame [28].

The loss of an open reading frame implies that such rodent-specific unitary pseudogenes no longer encode a functional protein and that, if transcribed, conservation of their transcriptional or post-transcriptional regulatory mechanisms is independent of their ancestral coding function. We have shown that despite their loss of protein-coding potential, the ancestral tissue expression patterns and gene expression levels show tendencies to be retained in the rodent transcribed unitary pseudogenes. We have presented evidence that the preservation of ancestral post-transcriptional networks is due, at least in part, to the retention of MREs within competitive endogenous RNA transcripts.

Our analysis of naturally occurring hypomorphs (namely, unitary pseudogenes) has allowed us to infer the relative importance of non-protein to protein encoded functions of a set of mRNAs. For human orthologs of 17 rodent transcribed unitary pseudogenes we have provided evidence that their miRNA decoy functions are unlikely to be subordinate to their protein-coding functions. We tested and validated this prediction using *BCAS4*, an exemplar of one such transcribed unitary pseudogenes. Instead, it appears that the post-transcriptional regulatory roles of some loci can outlive their protein-coding functions, and are sufficiently important for selection against deleterious mutations to maintain their transcription.

Transcription of duplicated and unitary pseudogenes in eukaryotes has been previously proposed to argue for their functionality [41]. A few anecdotal examples have demonstrated that new noncoding functions can arise from pseudogenized protein-coding loci [42,43]. A well-known example of such loci is *Xist*, which arose in eutherians by pseudogenization of a pre-existing protein-coding gene [44] and is essential for X-chromosomal inactivation in early developmental stages in mammalian females [43]. These unitary pseudogenes appear to retain the functions, namely their post-transcriptional miRNA-dependent roles, of their orthologous protein-coding ancestors. It remains unknown whether transcribed pseudogenes more frequently evolve new functions, or retain, at least in part, their preexisting functions. Similar questions have been asked of the evolution of duplicated genes [44], although the lack of duplication of these transcribed unitary pseudogenes has clearly prohibited the partitioning of both ancestral functions.

Our conclusions are based on the analysis of unitary pseudogenes that arose in the common ancestor of rodents. We note that unitary pseudogenes in other mammalian lineages have also been shown to be often transcribed and that crosstalk between transcripts that share MREs has been described in diverse eukaryotic lineages. Consequently, we hypothesize that transcription of expressed unitary pseudogenes led to the preservation of auxiliary post-transcriptional regulatory roles of bifunctional mRNAs.

## Materials and methods

### Annotation of unitary pseudogenes in mouse

Transmap annotations of human mRNAs in the mouse, rat and dog genomes were downloaded from UCSC [45]. To remove misannotations, only protein-coding genes that were also annotated in ENSEMBL build 67 [46] were considered for the remainder of the analysis. Exonerate [31] was used to produce exhaustive 6-frame pairwise alignments between human polypeptides and predicted syntenic regions (plus 5 kb flanking sequences) of mouse, rat and dog genome assemblies. The best pairwise alignment was classified according to the criteria defined elsewhere [32] as representing either a conserved protein-coding gene or a unitary pseudogene. Pairwise alignments between human polypeptides that appeared conserved in dog, but lost in both mouse and rat, were visually inspected to ensure that loss of function mutations arose prior to the last common ancestors of mouse and rat, and that the gene order ('conserved synteny') for the loci flanking the putative rodent-specific loss was conserved across all four mammalian species.

### Gene expression and transcript assembly

Publicly available polyA-selected RNA singlend sequencing reads for mouse and human adult tissues (testis, liver, heart, kidney, brain and cerebellum) from the study of Brawand and colleagues [35] were downloaded from the Short Read Archive [47]. Reads were aligned to the corresponding reference genome using Tophat [48]. A file containing the mapped coordinates of mouse and rat ESTs and mRNA mapped coordinates (downloaded from UCSC [45] on 11 March 2011) was provided to Tophat to facilitate its mapping of reads across splice junctions. Unitary pseudogenes whose predicted genomic location [28] overlapped by at least one sequencing read in at least one tissue were considered to be expressed. For each tissue in mouse, reads mapping to rRNA, tRNA and mitochondrial RNA were masked and the remainder used to assemble, using Cufflinks [36], transcripts *de novo *across the predicted genomic location of unitary pseudogenes. A reference annotation was produced by combining transcripts assembled in the different tissues using Cuffcompare [36]. Mouse transcripts were also assembled using stranded paired-end polyA-selected RNA sequencing reads from the study of Shen and colleagues [37]. Mapped reads for all 19 available tissues and cell lines were downloaded from the Gene Expression Omnibus [49] and assembled, using Cufflinks [36], transcripts *de novo *across the predicted genomic location of unitary pseudogenes. A reference annotation was produced by combining transcripts assembled in the different tissues using Cuffcompare [36].

Mouse and human protein-coding transcript annotations were downloaded from ENSEMBL (build 67) [50]. Total numbers of reads overlapping protein-coding gene constitutive exons or expressed nucleotides within unitary pseudogenes were normalized using TMM (edgeR package) [51]. Briefly, to estimate the normalized library size for each species, it was assumed that 60% of expressed genes were transcribed at similar levels in the two species. The normalized mouse and human library size was used to calculate the expression level (as FPKM) of each locus in each tissue in both species.

We estimated the median expression and tissue specificity across the six mouse adult tissues. We calculated tissue specificity (T*_S_*) values for each tissue and each locus. T*_S _*is defined as the fractional expression of a locus in one tissue relative to the sum of its expression in all tissues. The maximum T*_S _*value (maxT*_S_*) for a locus thus provides an indicator of tissue specificity, with higher values reflecting more tissue-specific expression [52].

### Protein-coding potential of unitary pseudogenes

We calculated and compared the PhyloCSF score [38] for mouse-rat pairwise alignments of transcribed unitary pseudogenes, 1,000 randomly selected protein-coding gene transcripts fragments with a matching size distribution. We also used Coding Potential Calculator [39] to estimate the coding potential of transcribed unitary pseudogenes and 1,000 randomly selected protein-coding gene transcript fragments with a matching size distribution.

### Expression correlation

The Pearson correlation coefficient between expression values across six tissues for a mouse unitary pseudogene and its human protein-coding ortholog was computed. For comparison, the correlation in expression between non-orthologous pairs of randomly selected protein-coding genes and pairs of mouse-human one-to-one orthologs was also estimated. Mouse-human one to one orthologs were downloaded from ENSEMBL [53].

To identify protein-coding genes whose tissue expression is significantly correlated with those of mouse unitary pseudogenes, the Pearson correlation in expression across mouse tissues was calculated for all possible pairs of unitary pseudogene and protein-coding gene loci. Only mouse protein-coding genes with a one-to-one orthologous relationship with genes in the human genome were considered. The associated *P*-value for each correlation was compared to the distribution of *P*-values associated with the correlation of 10,000 pairs of randomized expression vectors. A similar analysis was performed for all human protein-coding orthologs of mouse unitary pseudogenes.

### Prediction of miRNA response elements

The sequences of 3' UTRs of all mouse and human protein-coding genes were downloaded from UCSC [34]. Sequences of mouse and human miRNA families were downloaded from the TargetScan website (August 2010 version) [54]. Only families conserved between mouse and human were considered in the remainder of the analysis. TargetScan (TargetScan_50) was used to predict MREs in mouse and human 3' UTRs and transcribed regions of mouse unitary pseudogenes.

### 5' and 3' RACE

Total RNA from N2A cells was extracted using the RNAeasy kit (Qiagen, United Kingdom) followed by DNAse treatment with the DNA-free kit (Ambion, United Kingdom), according to the manufacturer's instructions. cDNA was prepared using a RACE ready cDNA kit (Clontech, France). PCR amplifications were carried out using primers specific to the 5' and 3' ends of the transcript and 5' and 3' RACE outer primers provided by the manufacturer. PCR reaction products were further amplified using nested sequence primers and 5' and 3' RACE inner primers. The resulting product was purified using PCR cleanup kit (Qiagen), cloned into a TOPO vector (Invitrogen, United Kingdom) and sequenced.

### *Pbcas4 *knockdown

Small interfering RNAs specific to *Pbcas4 *were designed using the small interfering RNA selection program from the Whitehead Institute. As control we randomly permutated nucleotides and chose one oligo (Additional file 9) that had no significant similarity to mRNAs in the mouse genome. Designed small interfering RNAs and scramble control sequences were reverse complemented and the two arms of the hairpin linked by a loop sequence (TTCAAGAGA). Adapters required for cloning were added and the custom made oligos purchased from Sigma-Aldrich (United Kingdom). The HPLC purified oligos were resuspended in water to a final concentration of 100 μM. For each shRNA, 10 μl of forward and reverse oligos were added to 160 μl of annealing buffer (10 mM Tris pH 8, 50 mM of NaCl) and incubated for 5 minutes at 95°C. After cooling to room temperature oligos were phosphorylated using T4 polynucleotide kinase enzyme (New England BioLabs, United Kingdom) and cloned downstream of a U6 promoter from a modified pll3.7 vector (courtesy of Dr Esther Becker).

N2A cells were grown under standard conditions and 24 h before transfection, 1 × 10^5 ^cells/well were plated in six-well cluster culture vessels. Transient transfection of 4 μg of shRNA constructs and scramble control was carried out using Lipofectamine Plus (Invitrogen) in triplicate. Cells were harvested 72 h post-transfection and their RNA extracted using an RNAeasy kit (Qiagen) followed by DNAse treatment with the DNA-free kit (Ambion), according to the manufacturer's instructions. cDNA was prepared as described above and used to assay *Pbcas4 *down-regulation using quantitative PCR. RNA was used for mRNA expression profiling (below). Transfections of *Pbcas4*-shRNA led to a reproducible 50% decrease in expression of this unitary pseudogene in N2A cells.

### mRNA expression profiling

RNA integrity was assessed on a BioAnalyzer; all samples had an RNA Integrity Number (RIN) ≥ 7 (Agilent Laboratories, US). Sense single-stranded DNA was generated from 200 ng starting RNA with the Ambion^® ^WT Expression Kit according to the manufacturer's instructions and fragmented and labeled using the GeneChip^® ^WT Terminal Labeling and Controls Kit. The distribution of fragment lengths was measured on a BioAnalyser. The labeled single-stranded DNA was hybridized to the Affymetrix Mouse Gene 1.0 ST Array (Affymetrix). Chips were processed on an Affymetrix GeneChip Fluidics Station 450 and Scanner 3000. Cel files were generated using Command Console (Affymetrix). Limma, from the bioconductor package, was used to identify differentially expressed genes (Benjamini-Hochberg corrected *P*-value < 0.05) between *Pbcas4 *and scramble vector transfected cells. We considered only probes where variance between conditions exceeded 0.5.

These data are accessible through Gene Expression Omnibus accession number GSE38333.

### Contribution of miRNA binding

Out of 165 genes whose expression was significantly changed upon *Pbcas4 *knock-down in mouse, 84 had a one-to-one orthologous gene in human whose correlation to *BCAS4 *could be determined. Out of the 57 human orthologs of significantly down-regulated mouse genes, 41 were positively correlated in expression with *BCAS4*; 28 would be expected by chance. For comparison a similar analysis was performed for genes significantly up-regulated upon *Pbcas4 *knockdown. Out of the 26 human orthologs, 16 were positively correlated in expression with *BCAS4*. This is not a significant deviation from the 13 that would be expected by chance (*P *= 0.3, binomial test).

### Validation of post-transcriptional regulation by miR-185

N2A and SH-SY5Y cells were prepared 24 h before transfection as described above ('*Pbcas4 *knockdown'). miR-185 mimics and negative control miRNA mimic (50 nM; Applied Biosystems, United Kingdom) were transfected using Lipofectamine^® ^RNAiMAX Reagent (Invitrogen). Cells were harvested 24 h post-transfection. RNA was extracted as previously described. Mature miR-185 was reversed transcribed and quantified, following the manufacturer's instructions, using the TaqMan^® ^MicroRNA Reverse Transcription Kit and Taqman^® ^MicroRNA Assays (Applied Biosystems). Expression level of miR-185 was normalized to 18S rRNA. mRNA expression was detected as described above ('*Pbcas4 *knockdown').

### Statistics

Fisher's exact and Mann-Whitney tests were performed using the R package [55].

## Abbreviations

ceRNA: competitive endogenous RNA; FPKM: fragments per kilobase of sequence per million reads mapped; maxT*_S_*: maximum tissue specificity; miRNA: microRNA; MRE: microRNA response element; RACE: rapid amplification of cDNA ends; RT-PCR: reverse transcription polymerase chain reaction; shRNA: short hairpin RNA; T*_S_*: tissue specificity; UTR: untranslated region.

## Competing interests

The authors declare that they have no competing interests.

## Authors' contributions

ACM designed the study and identified and characterized *in silico *and *in vitro *unitary pseudogenes in mouse. LK and AH provided support on modifications to the Gpipe gene prediction pipeline. SL performed gene expression profiling. JT experimentally tested the conserved miRNA decoy function of *Pbcas4*. ACM and CPP wrote the manuscript. All authors read and approved the final manuscript.

## Supplementary Material

Additional file 1 **Rodent-specific unitary pseudogenes**. Click here for file

Additional file 2 **Human CPO peptide complete pairwise alignments**. Click here for file

Additional file 3 **Mouse unitary pseudogene transcripts (mm9) using **[35]. Click here for file

Additional file 4 **Mouse unitary pseudogene transcripts (mm9) using **[37]. Click here for file

Additional file 5 **Codon substitution pattern of unitary pseudogenes**. The coding substitution pattern unitary pseudogene (green) is significantly smaller (***P < 0.001) than that of protein-coding transcript fragments (blue) with matching size. Only transcripts with a sequence allowing reliable prediction of an open reading frame (94 and 722 unitary pseudogenes and protein-coding transcripts, respectively) were considered. Click here for file

Additional file 6 **Unitary transcribed pseudogene and protein-coding expression**. **(a, b) **Median normalized expression (log 2 fragments per kilobase of exon per million read) (a) and maximum tissue specificity (maxT*_S_*) (b) across six mouse adult tissue unitary transcribed pseudogenes (green) and protein-coding genes (blue). Click here for file

Additional file 7 **Expression level and tissue specificity of transcribed unitary pseudogenes**. Click here for file

Additional file 8 **Tissue expression correlation between mouse and human loci**. Distribution of mouse-human expression correlation (Pearson) between 1,000 mouse-human random pairs of non-orthologous protein-coding genes (grey) and mouse unitary pseudogene protein-coding orthologs (green). The *P*-value associated with the comparison between these distributions is 0.23. Click here for file

Additional file 9 **Custom oligonucleotide sequences**. Click here for file

Additional file 10 **Selected protein-coding candidates for validation of miR-185 binding in mouse and humans**. Click here for file
