# Supplementary material for: Evidence for conserved post-transcriptional roles of unitary pseudogenes and for frequent bifunctionality of mRNAs
Source: Genome Biol. 2012 Nov 15;13(11):R102. doi: 10.1186/gb-2012-13-11-r102 (PMC3580494; doi:10.1186/gb-2012-13-11-r102)
Supplement: Additional file 2 — Human CPO peptide complete pairwise alignments. [file gb-2012-13-11-r102-S2.PDF]

a. Dog

```
    1 : MetLysProLeuLeuGluThrLeuTyrLeuLeuGlyMetLeuValProGlyGlyLeuGly :    20
        |||||||||||||||! !||||||! !|||||||||!!:|||:!!||||! |||||||||
        MetLysProLeuLeuGlyThrLeuCysLeuLeuGlyIleLeuIleProGluGlyLeuGly
 5052 : ATGAAGCCTCTGCTTGGGACCCTTTGTCTTTTGGGGATCCTGATTCCTGAGGGGCTGGGA :  5109

   21 : TyrAsp{Ar}  >>>> Target Intron 1 >>>>  {g}SerLeuAlaGlnHisArg :    29
        ||||||{ !}          10329 bp          {!}||||||||||||! ||||
        TyrAsp{Gl}++                        ++{y}SerLeuAlaGlnProArg
 5110 : TATGAT{GG}gt........................ag{A}TCCCTAGCACAACCCAGA : 15465

   30 : GlnGluIleValAspLysSerValSerProTrp<->SerLeuGluThrTyrSerTyrAsn :    48
        !::|||:!!||||!!::!!!!!! !|||||||||   ||||||!!:||||||||||||!.!
        ArgGluValValGluGluThrGlySerProTrpGlnSerLeuAspThrTyrSerTyrThr
15466 : CGTGAGGTTGTGGAAGAGACAGGGAGCCCGTGGCAGAGCCTGGACACCTATTCCTATACC : 15525

   49 : IleTyrHisProMetGlyGlu  >>>> Target Intron 2 >>>>  IleTyrGluT :    59
        ! !|||||||||||||! ||||           2067 bp          ||||||:!!|
        ArgTyrHisProMetGluGlu++                        ++IleTyrGlnT
15526 : AGATATCACCCTATGGAGGAGgt........................agATCTATCAGT : 17625

   60 : rpMetArgGluIleSerGluLysTyrLysGluValValThrGlnHisPheLeuGlyValT :    79
        |||||! !:!!||||||||||||||||  !! !||||!.!||||||||||||||||||:!!|
        rpMetIleGlnIleSerGluLysTyrAlaGlyValAlaThrGlnHisPheLeuGlyMetT
17626 : GGATGATCCAGATAAGTGAGAAGTACGCAGGAGTGGCGACACAGCATTTCCTAGGCATGA : 17685

   80 : hrTyrGluThrHisProMetTyrTyrLeuLys  >>>> Target Intron 3 >>>>  :    90
        |||||||||||!..||||||||||||||||||           1296 bp
        hrTyrGluThrArgProMetTyrTyrLeuLys++                         +
17686 : CCTATGAGACCCGGCCCATGTATTATTTGAAGgt.........................a : 19012

   91 :  IleSerGlnProSerGlyAsnProLysLysIleIleTrpMetAspCysGlyIleHisAl :   109
         |||||||||||||||! !||||||||||||||||||||||||||||||||||||||||||
        +IleSerGlnProSerAspAsnProLysLysIleIleTrpMetAspCysGlyIleHisAl
19013 : gATCAGCCAGCCATCCGATAACCCCAAGAAGATCATTTGGATGGACTGTGGAATTCATGC : 19071

  110 : aArgGluTrpIleAlaProAlaPheCysGlnTrpPheValLysGlu  >>>> Target  :   125
        ||||||||||||||||||||||||||||||||||||||||||||||             87
        aArgGluTrpIleAlaProAlaPheCysGlnTrpPheValLysGlu++
19072 : CAGGGAATGGATTGCCCCAGCTTTTTGCCAATGGTTTGTGAAAGAAgt............ : 19121

  126 : Intron 4 >>>>  IleLeuGlnAsnHisLysAspAsnSerSerIleArgLysLeuLeu :   139
        1 bp           ||||||||||||:!!|||||||||| !!!!!||| !!!:!!!!|||
                     ++IleLeuGlnAsnTyrLysAspAsnProArgIleGlyArgPheLeu
19122 : .............agATTCTACAAAACTATAAAGACAACCCAAGGATAGGAAGATTCCTT : 20032

  140 : ArgAsnLeuAspPheTyrValLeuProValLeuAsnIleAspGlyTyrIleTyrThrTrp :   159
        ||||||||||||||||||||||||||||||||||||||||||||||||||||||||||||
        ArgAsnLeuAspPheTyrValLeuProValLeuAsnIleAspGlyTyrIleTyrThrTrp
20033 : AGAAACCTGGACTTCTATGTGCTTCCAGTTCTTAATATAGATGGTTATATCTATACTTGG : 20092

  160 : ThrThr  >>>> Target Intron 5 >>>>  AspArgLeuTrpArgLysSerArgS :   170
        ||||||          1124 bp           |||||||||||||||||||||||||
        ThrThr++                        ++AspArgLeuTrpArgLysSerArgS
20093 : ACAACTgt........................agGATAGGCTTTGGAGGAAATCTCGTT : 21249

  171 : erProHisAsnAsnGlyThrCysPheGlyThrAspLeuAsnArgAsnPheAsnAlaSerT :   190
        || !!|||||||||||||||||||||||||||||||||||||||||||||||!.!||||
        erSerHisAsnAsnGlyThrCysPheGlyThrAspLeuAsnArgAsnPheAsnValSerT
21250 : CATCCCATAATAACGGCACATGTTTTGGGACAGATCTCAATCGAAATTTCAACGTTTCCT : 21309

  191 : rpCys{S}  >>>> Target Intron 6 >>>>  {er}IleGlyAlaSerArgAsnC :   199
        |||||{|}          1142 bp          {||}|||||||||||||!:!||||
        rpCys{S}++                        ++{er}IleGlyAlaSerLysAsnC
21310 : GGTGT{A}gt........................ag{GT}ATTGGCGCATCGAAAAACT : 22478

  200 : ysGlnAspGlnThrPheCysGlyThrGlyProValSerGluProGluThrLysAlaValA :   219
        ||||||||  !|||||||||||||:!!||||||||||||||||||||||||||||||||:
        ysGlnAspIleThrPheCysGlySerGlyProValSerGluProGluThrLysAlaValS
22479 : GCCAGGATATAACATTCTGTGGCTCAGGACCAGTGTCAGAACCAGAGACTAAAGCCGTTT : 22538

  220 : laSerPheIleGluSerLysLysAspAspIleLeuCysPheLeuThrMetHisSerTyrG :   239
        !!||||||:!:||||||||||||!!:..!|||:!!|||||||||||||||||||||||||
        erSerPheValGluSerLysLysGluSerIleValCysPheLeuThrMetHisSerTyrG
22539 : CCAGCTTTGTGGAGAGCAAGAAGGAGAGCATCGTGTGCTTCCTGACCATGCACTCCTACG : 22598

  240 : lyGlnLeuIleLeuThrProTyrGlyTyrThrLysAsnLysSerSerAsnHisProGluM :   259
        |||||||||||||||||||||||||||||||||||||||||||||||||||  !|||:
        lyGlnLeuIleLeuThrProTyrGlyTyrThrLysAsnLysSerSerAsnHisGluGluL
22599 : GGCAGCTCATCCTCACGCCTTATGGCTACACGAAAAATAAGTCCAGTAACCACGAGGAAC : 22658

  260 : et  >>>> Target Intron 7 >>>>  IleGlnValGlyGlnLysAlaAlaAsnAl :   269
        !!          5765 bp           |||||||||||||||||||||||||||||
        eu++                        ++IleGlnValGlyGlnLysAlaAlaAsnAl
22659 : TGgt........................agATCCAAGTTGGACAGAAGGCAGCAAATGC : 28453

  270 : aLeuLysAlaLysTyrGlyThrAsnTyrArgValGlySerSerAlaAspIleLeu{T}   :   288
        |||||||  !|||:!!||||||||||||||||||||||||||||||||||||||||{|}
        aLeuLysLysLysHisGlyThrAsnTyrArgValGlySerSerAlaAspIleLeu{T}++
28454 : ATTGAAAAAAAAGCATGGAACCAATTATAGAGTTGGATCGAGTGCAGATATTTTA{T}gt : 28511

  289 : >>>> Target Intron 8 >>>>  {yr}AlaSerSerGlySerSerArgAspTrpAl :   298
                  863 bp          {||}|||!!!||||||||||||||||||||||
                            ++{yr}AlaThrSerGlySerSerArgAspTrpAl
28512 : ........................ag{AT}GCCACATCAGGGTCTTCAAGAGACTGGGC : 29403

  299 : aArgAspIleGlyIleProPheSerTyrThrPheGluLeuArgAspSerGlyThrTyrGl :   318
        |||||||||||||||||||||||||||||||||||||||||||||!:!||||||||||||
        aArgAspIleGlyIleProPheSerTyrThrPheGluLeuArgAspAsnGlyThrTyrGl
29404 : TCGGGACATTGGGATCCCCTTCTCATACACATTTGAGCTCCGGGACAATGGTACATATGG : 29463

  319 : yPheValLeuProGluAlaGlnIleGlnProThrCysGluGluThrMetGluAlaValLe :   338
        ||||:!!||||||||! !|||||||||||| !!|||||||||||||||||||||||||||
        yPheIleLeuProAlaAlaGlnIleGlnAlaThrCysGluGluThrMetGluAlaValLe
29464 : ATTTATTTTGCCAGCAGCTCAGATTCAGGCCACCTGCGAGGAGACCATGGAGGCTGTGCT : 29523

  339 : uSerValLeuAspAspValTyrAlaLysHisTrpHisSerAspSerAlaGlyArgValTh :   358
        |||||||||||||||||||||! !|||:!!|||:!!|||||||||||||||!:!||||||
        uSerValLeuAspAspValTyrGluLysTyrTrpAsnSerAspSerAlaGlyLysValTh
29524 : CTCGGTCCTGGATGATGTATATGAGAAATACTGGAATTCAGACAGTGCTGGCAAGGTGAC : 29583

  359 : rSerAlaThr :   361
        | !!||||||
        rProAlaThr
29584 : ACCTGCCACG : 29594
```

b. Mouse

```
  223 : GluSerLysLysAspAspIleLeuCysPheLeuThrMetHisSerTyrGlyGlnLeuIle :   242
        :::...::::::     !.!!||||||||||||||||! !!.!!!!|||||||||||||||||
        LysLysGluArgLysCysPheLeuCysPheLeuThrThrArgCysTyrGlyGlnLeuIle
29821 : aagaaagaaagaaagTGCTTCCTGTGCTTCCTGACCACGCGCTGTTATGGGCAGCTCATT : 29878

  243 : LeuThrProTyrGlyTyrThrLysAsnLysSerSerAsnHisProGluMet  >>>> Ta :   260
        ||||||||||||||||||||||||||||||!!|||||!!.:!!  !.!.:!:
        LeuThrProTyrGlyTyrThrLysAsnLysThrSerLysTyrGluHisLeu++
29879 : CTCACACCATATGGCTACACCAAAAATAAAACAAGTAAATACGAGCATCTAgt....... : 29934

  261 : rget Intron 1 >>>>  IleGlnValGlyGlnLysAlaAlaAsnAlaLeuLysAlaL :   273
          5702 bp          |||||||||||||||||||||||||!:!.!!||||||||||||
                          ++IleGlnValGlyGlnLysAlaAlaSerThrLeuLysAlaL
29935 : ..................agATTCAAGTTGGACAGAAGGCGGCAAGCACACTGAAAGCAA : 35673

  274 : ysTyrGlyThrAsnTyrArgValGlySerSerAlaAspIleLeu{T}  >>>> Target :   288
        ||:!!|||||||||||||||||||||||||||||||||||||||{|}              8
        ysHisGlyThrAsnTyrArgValGlySerSerAlaAspIleLeu{T}++
35674 : AGCACGGAACCAATTATAGAGTTGGATCGAGTGCAGATATTTTA{T}gt........... : 35721

  289 :  Intron 2 >>>>  {yr}Ala-SerSerGlySerSerArgAspTrpAlaArgAspIle :   301
        95 bp           {||}|||#   ||||||||||||!:!|||||||! ! !!||||||
                      ++{yr}Ala#---SerGlySerSerLysAspTrpAspGlyAspIle
35722 : ..............ag{AT}GCTG---TCAGGATCCTCAAAAGACTGGGATGGAGACATT : 36650

  302 : GlyIleProPheSerTyrThrPheGluLeuArgAspSerGlyThrTyrGlyPheValLeu :   321
        |||||| !!||||||||||||||||||||||! !||||!:!|||||||:!!||||||||!.!|||
        GlyIleSerPheSerTyrThrPheGluLeuMetAspAsnGlyThrHisGlyPheAlaLeu
36651 : GGGATCTCCTTCTCCTACACATTTGAGCTAATGGACAATGGTACCCATGGGTTTGCTCTG : 36710

  322 : ProGluAlaGlnIleGlnProThrCysGluGluThrMetGluAlaValLeuSerValLeu :   341
         !! !!..!            ..!||||||||||.!!! !!!:|||:!!||||! |||||||
        Thr***Cys------------ValCysGluGluAlaLysAspAlaIleLeuLeuValLeu
36711 : ACCTAGTGC------------GTATGTGAAGAGGCCAAGGATGCCATTCTCTTAGTCCTT : 36758

  342 : AspAspValTyrAlaLysHisTrpHisSerAspSerAla :   354
        |||||||||||||! !!!.  !|||  !!!!:!!||||||
        AspAspValTyrGluAsnPheTrpSerThrAsnSerAla
36759 : GATGATGTTTATGAGAATTTCTGGTCCACCAACAGTGCC : 36799
```

c. Rat

```
  168 : SerArgSerProHisAsnAsnGlyThrCysPheGlyThrAspLeuAsnArgAsnPhe{A} :   187
        ||| !! !! !!|||:!!!:!! !||||||||||||||||||||||||| !! !!|||{!}
        SerGlyProSerHisHisSerAspThrCysPheGlyThrAspLeuAsn***TyrPhe{S}
27846 : TCAGGACCATCCCATCATAGTGACACATGCTTTGGAACAGATCTCAATTGATATTTC{A} : 27903

  188 :   >>>> Target Intron 1 >>>>  {sn}AlaSerTrpCys<->SerIleGlyAla :   195
                  1777 bp          {:!}  !  !!::! !   ..!||||||.!!
        ++                       ++{er}LeuLeuTyrPheProAspIleGlyThr
27904 : gt........................ag{GC}CTCCTGTATTTCCCAGATATCGGCACA : 29707

  196 : SerArgAsnCysGlnAspGlnThrPheCysGlyThrGlyProValSerGluProGluThr :   215
         !!! !|||||||||||  !|||||||||||||! !! !|||:!!! !|||||||||  !
        ProIleAsnCysGlnAspValThrPheCysGlyIleGluProMetLeuGluProGluLeu
29708 : CCCATAAACTGCCAAGATGTAACATTCTGTGGGATAGAACCAATGTTGGAACCAGAACTC : 29767

  216 : LysAlaValAlaSerPheIleGluSerLys----LysAspAspIleLeuCysPheLeuTh :   234
        !   !!  !  !:!!!!.   :!!:!!!:!####||| ! ||||||||||||||||||!
        ThrProSerGlnAlaLeuGlnLysAlaArg####LysLysAspIleLeuCysPheLeuIl
29768 : ACTCCTTCTCAAGCCTTACAGAAAGCAAGGGGGAAAAAAAGACATCCTGTGCTTCCTGAT : 29828

  235 : rMetHisSerTyrGlyGlnLeuIleLeuThrProTyrGlyTyrThrLysAsnLysSerSe :   254
        !|||  !||||||||||||||||||||||||||||||||||:!!|||||||||||| !!
        eMetGlySerTyrGlyGlnLeuIleLeuThrProTyrGlyHisThrLysAsnLysProHi
29829 : CATGGGCTCTTATGGGCAACTCATTCTCACACCATATGGCCACACCAAAAATAAACCACA : 29888

  255 : rAsnHisProGluMet  >>>> Target Intron 2 >>>>  IleGlnValGlyGln :   264
        !|||:!!  !|||:!:           5837 bp           |||||||||||||||
        sAsnTyrGluGluLeu++                        ++IleGlnValGlyGln
29889 : TAACTACGAAGAACTAgt........................agATTCAAGTTGGACAG : 35755

  265 : LysAlaAlaAsnAlaLeuLysAlaLysTyrGlyThrAsnTyrArgValGlySerSerAla :   284
        |||||||||!..||||||||||||||:!!||||||||||||||||||||||||||.!!||||
        LysAlaAlaArgAlaLeuLysAlaLysHisGlyThrAsnTyrArgValGlySerGlyAla
35756 : AAGGCGGCAAGAGCCCTGAAAGCAAAGCATGGAACCAATTATAGAGTTGGATCGGGTGCA : 35815

  285 : AspIleLeu{T}  >>>> Target Intron 3 >>>>  {yr}AlaSerSerGlySer :   293
        |||||||||{|}           855 bp           {||}|||  !!!!  !  !
        AspIleLeu{T}++                        ++{yr}AlaValArgIleLeu
35816 : GATATTTTA{T}gt........................ag{AT}GCTGTCAGGATCCTC : 36697

  294 : SerArg<->AspTrpAlaArgAspIleGlyIleProPheSerTyrThrPheGluLeuArg :   312
        !.!|||   !!:|||  !!.!     !  !:!!! !||||||||||||||||||||  !
        LysArgLeuGluTrpArgHisTrpAspProLeuLeuPheSerTyrThrPheGluLeuVal
36698 : AAAAGACTGGAATGGAGGCATTGGGATCCCCTTCTCTTCTCATACACATTTGAGCTGGTG : 36757

  313 : AspSerGlyThrTyrGlyPheValLeu :   321
        |||!:!||||||:!!||||||||!.!|||
        AspAsnGlyThrHisGlyPheAlaLeu
36758 : GACAATGGTACCCATGGGTTTGCTCTG : 36786
```
