# Supplementary figures and images for: Evidence for conserved post-transcriptional roles of unitary pseudogenes and for frequent bifunctionality of mRNAs
Source: Genome Biol. 2012 Nov 15;13(11):R102. doi: 10.1186/gb-2012-13-11-r102 (PMC3580494; doi:10.1186/gb-2012-13-11-r102)

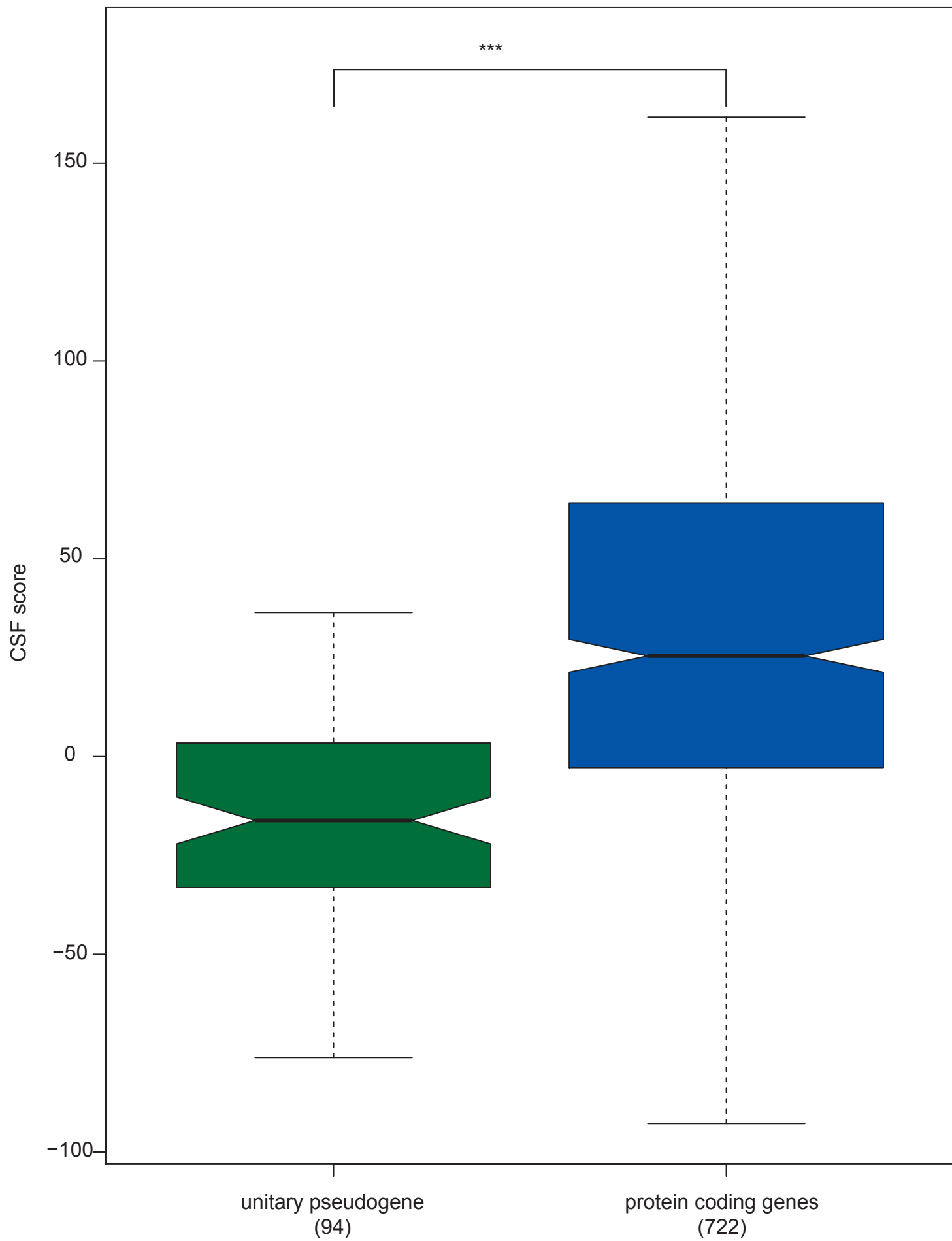

Supplement: Additional file 5 — Codon substitution pattern of unitary pseudogenes. The coding substitution pattern unitary pseudogene (green) is significantly smaller (***P < 0.001) than that of protein-coding transcript fragments (blue) with matching size. Only transcripts with a sequence allowing reliable prediction of an open reading frame (94 and 722 unitary pseudogenes and protein-coding transcripts, respectively) were considered. [file gb-2012-13-11-r102-S5.PDF]

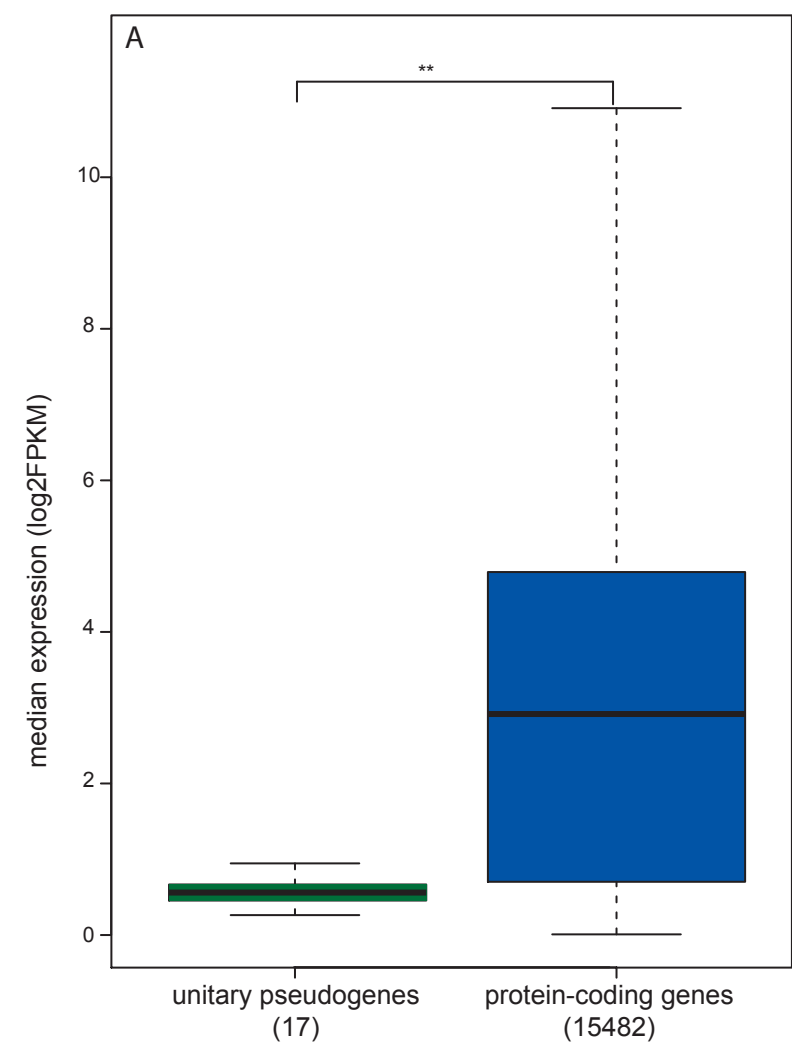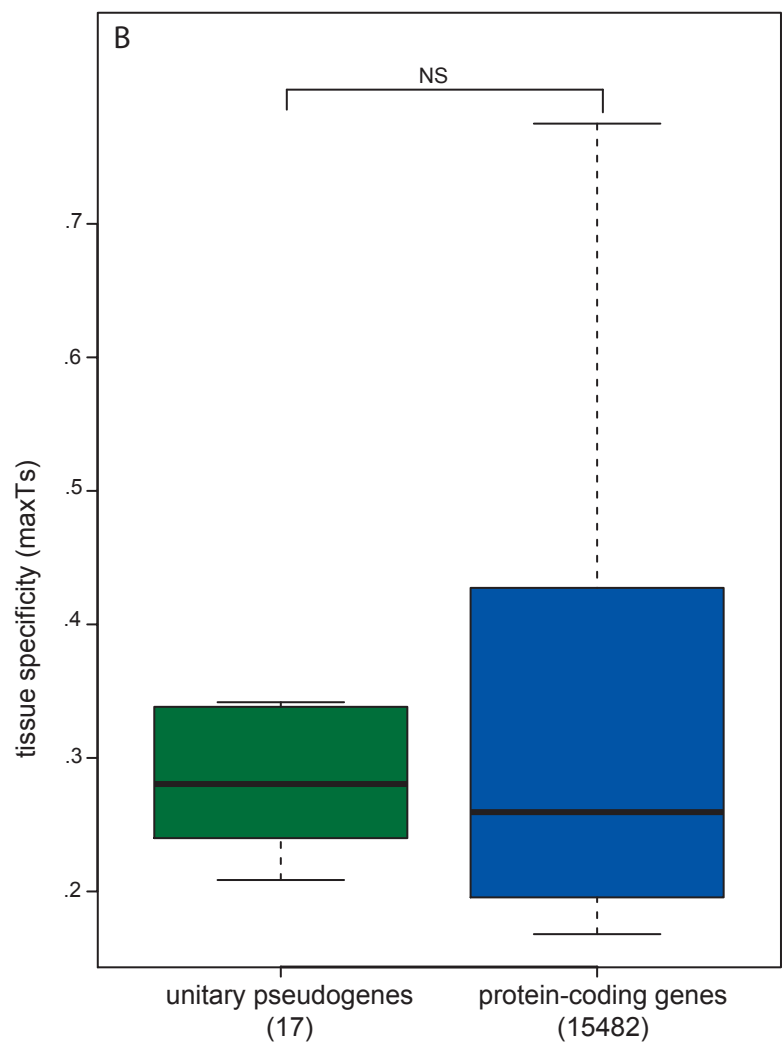

Supplement: Additional file 6 — Unitary transcribed pseudogene and protein-coding expression. (a, b) Median normalized expression (log 2 fragments per kilobase of exon per million read) (a) and maximum tissue specificity (maxTS) (b) across six mouse adult tissue unitary transcribed pseudogenes (green) and protein-coding genes (blue). [file gb-2012-13-11-r102-S6.PDF]

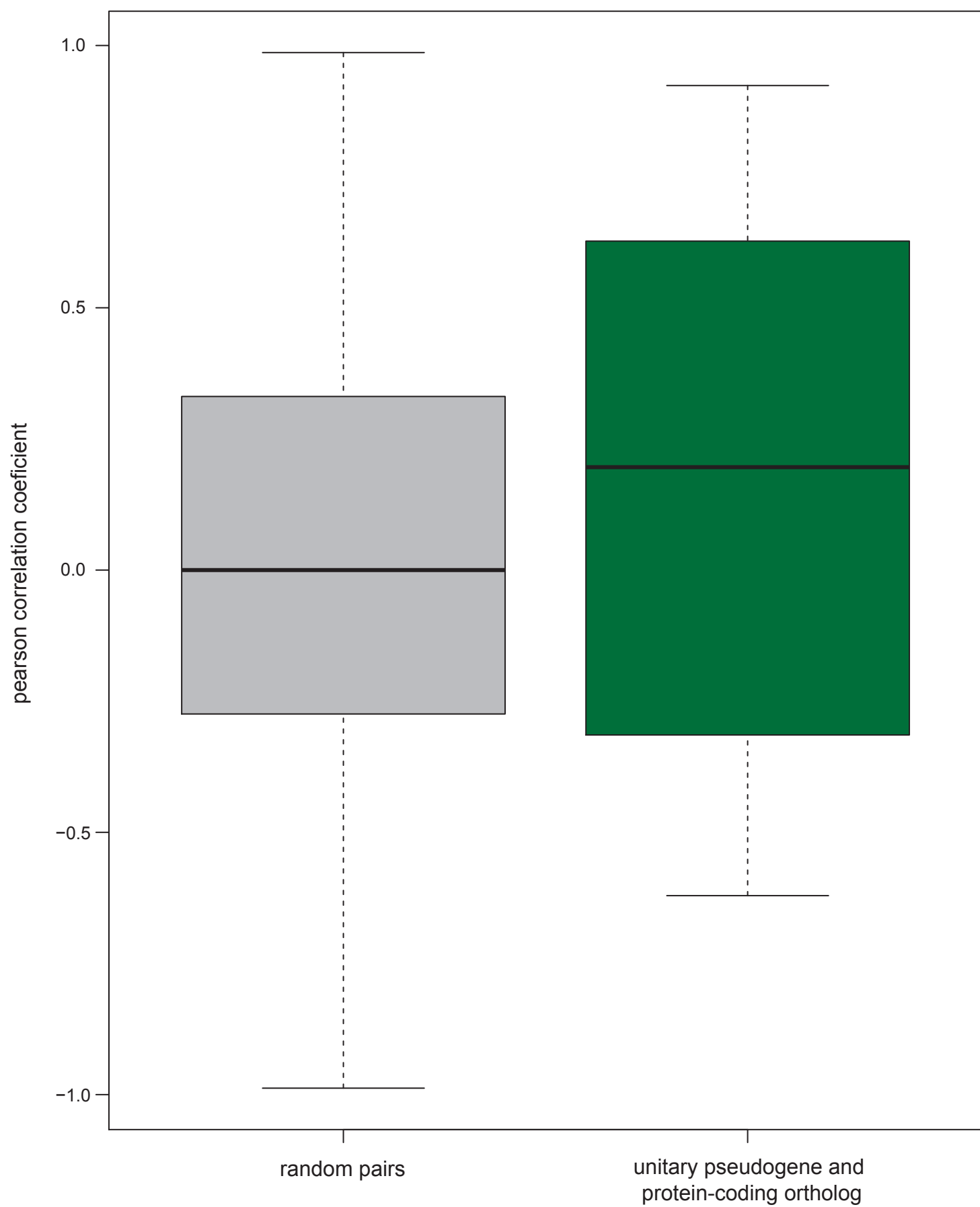

Supplement: Additional file 8 — Tissue expression correlation between mouse and human loci. Distribution of mouse-human expression correlation (Pearson) between 1,000 mouse-human random pairs of non-orthologous protein-coding genes (grey) and mouse unitary pseudogene protein-coding orthologs (green). The P-value associated with the comparison between these distributions is 0.23. [file gb-2012-13-11-r102-S8.PDF]
